# Physico-Mechanical and Biological Durability of Citric Acid-Bonded Rubberwood Particleboard

**DOI:** 10.3390/polym13010098

**Published:** 2020-12-29

**Authors:** Zhou Huaxu, Lee Seng Hua, Paridah Md Tahir, Zaidon Ashaari, Syeed SaifulAzry Osman Al-Edrus, Nor Azowa Ibrahim, Luqman Chuah Abdullah, Siti Fatahiyah Mohamad

**Affiliations:** 1 Institute of Tropical Forestry and Forest Products, Universiti Putra Malaysia (UPM), UPM Serdang 43400, Selangor, Malaysia; annazhouhuaxu@gmail.com (Z.H.); chuah@upm.edu.my (L.C.A.); fatahiyah@nuclearmalaysia.gov.my (S.F.M.); 2 Faculty of Forestry and Environment, Universiti Putra Malaysia (UPM), UPM Serdang 43400, Selangor, Malaysia; zaidon@upm.edu.my; 3 Faculty of Science, Universiti Putra Malaysia (UPM), UPM Serdang 43400, Selangor, Malaysia; 4 Faculty of Engineering, Universiti Putra Malaysia (UPM), UPM Serdang 43400, Selangor, Malaysia; 5 Radiation Processing and Technology Division, Malaysia Nuclear Agency, Bangi 43000, Selangor, Malaysia

**Keywords:** citric acid, particleboard, rubberwood, FTIR, white rot fungus, termites

## Abstract

This study investigated the effects of different citric acid content on the physico-mechanical and biological durability of rubberwood particleboard. Particleboards with density of 700 kg/m^3^ were produced with three different citric acid contents, namely 10, 15 and 20 wt%. Particleboards made from 10 wt% urea formaldehyde (UF) resin were served as control for comparison purposes. FTIR analysis was carried out and the formation of ester linkages between -OH on cellulose and carbonyl groups of citric acid was confirmed. The peak intensity increased along with increasing citric content, which indicated that a higher amount of ester linkages were formed at higher citric acid content. Citric acid-bonded particleboard had inferior physical properties (water absorption and thickness swelling) and mechanical properties (internal bonding strength, modulus of rupture and modulus of elasticity) compared to that of the UF-bonded particleboard. However, the performance of particleboard was enhanced with increasing citric acid content. Meanwhile, citric acid-bonded particleboard displayed significantly better fungal and termite resistance than UF-bonded particleboard owing to the acidic nature of citric acid. It can be concluded that citric acid is a suitable green binder for particleboard but some improvement is needed during the particleboard production process.

## 1. Introduction

Citric acid has been identified as an environmentally friendly treating agent and adhesive for wood and wood composites. Citric acid has been used in treating wood in order to confer the wood with better dimensional stability and biological durability [1,2]. Sefc et al. (2009) reported that beech (*Fagus sylvatica* L.) wood modified with 7.0% citric acid (CA) and 6.5% sodium hypophosphyte (SHP) as catalyst have higher resistance against brown rot fungus, *Poria placenta*. The resistance enhancement of citric acid modified beech wood could be attributed to the ester bonds between CA and beech wood [3]. Salem et al. [4] also stated that a combination of inner and outer bark extracts of sugar maple with citric acid could be a very effective biocide agent against molds. Generally, apart from enhanced dimensional stability and biology durability, wood modified with citric acid displayed improved modulus of elasticity (MOE) and compression strength and reduced water absorption [5]. Nevertheless, some drawbacks were also recorded, including reduction in both modulus of rupture and impact strength as well as the unfavourable yellowing after treatment [5].

As for wood binder, a review compiled by Lee et al. [5] revealed that citric acid has already been used as a main bonding component for a wide range of wood composites such as particleboard, plywood, wood-based molding, fibreboard and veneer-based panels. Kusumah et al. [6] bonded sweet sorghum bagasse particleboard with 20 wt% citric acid and pressed at temperature of 200 °C for 10 min. The author reported that the citric acid-bonded particleboard experienced lower mass loss when exposed to subterranean termite *Coptotermes formosanus* Shiraki compared to that of the control Sugi (*Cryptomeria japonica*) wood samples. In addition, higher termite mortality was also found in the citric acid-bonded particleboard where almost half of the termites were dead at the end of the test. Meanwhile, 24.57% termite mortality was found in the Sugi wood samples. Higher resistance against white rot and brown rot fungi was also observed in the citric acid-bonded particleboard compared to that of the Sugi wood samples. Interestingly, citric acid exhibited closely similar effectiveness in terms of termite and fungal resistance with polymeric 4,4′-methylenediphenyl isocyanate (pMDI). Another study by Indrayani et al. [7] reported that medium density fibreboard (MDF) fabricated from pineapple leaves bonded with citric acid showed better resistance against *C. formosanus* after 3-week exposure. However, until now, there have been very few researches reported on the resistance of citric acid-bonded particleboard against termites and fungal attack. 

In order to substitute formaldehyde-based resin that could pose serious threat to the end users, application of citric acid as main binding agent for wood and wood composite is encouraged. There are several factors that could affect the properties of the citric acid-bonded particleboard. The content of citric acid used is one of the vital parameters that is worth investigating [6]. Using the same resin dosage, particleboard bonded with citric acid may have inferior properties compared to that of particleboard bonded with formaldehyde-based resin. However, it is hypothesized that by increasing the citric acid content, the particleboard produced could exhibit comparable performance with the particleboard bonded with formaldehyde-based resins. Therefore, the objective of the present study is to investigate the effects of citric acid content on the physico-mechanical and biological durability of citric acid-bonded rubberwood particleboard and compared with urea formaldehyde-bonded particleboard.

## 2. Materials and Methods

### 2.1. Preparation of Materials

Rubberwood particles were obtained from HeveaBoard Berhad, a particleboard manufacturer located in Gemas, Negeri Sembilan. The obtained particles were dried to 3% in a laboratory oven prior to particleboard fabrication. Two main binders, namely urea formaldehyde (UF) and citric acid (CA), were used in this study for the fabrication of particleboard. UF resin with 60–65% solid content was purchased from Aica Chemicals (M) Sdn. Bhd located in Senawang. Meanwhile, CA in powder form was purchased from Evergreen Engineering & Resources, Semenyih, Selangor. The CA was dissolved in distilled water to achieve 60% solid content and used as binder for the particleboard production.

### 2.2. Production of Particleboard

Rubberwood (RW) particles were dried to 3% moisture content at 103 ± 2 °C prior to particleboard fabrication. Particleboard with the measurements of 340 mm × 340 mm × 12 mm was produced with the target density of 700 kg/m^3^. The rubberwood particles were blended with the citric acid at different dosages. The dosages used were 10, 15 and 20 wt% based on the oven dried weight of the rubberwood particles. Dosage of more than 20 wt% failed to form into particleboard due to excessive moisture of the sprayed particles. The control particleboard was fabricated by using urea formaldehyde (UF) with 10% resin dosage. Firstly, the rubberwood particles were placed in a blender where the binding agents was sprayed onto the particles during the blending process. After the blending process, the resinated wood particles were poured into a wooden mold to form a mat. Pre-press was applied to compact the mat. The mat was then be hot-pressed under 180 °C for 4.5 min with a pressure of 100 bar. Upon the completion of hot-pressing process, the particleboard formed was conditioned for 7 days in a conditioning room prior to evaluation of properties. Three boards were produced for each treatment variable. Thus, a total of 12 boards were produced.

### 2.3. Properties Evaluation 

#### 2.3.1. Fourier Transform Infrared (FTIR) Analysis

Rubberwood particles sprayed with different citric acid concentration were dried in an oven at 103 °C for 3 h before subjected to FTIR analysis. The FTIR measurements were conducted using a Perkin Elmer FTIR instrument (1 cm^−1^ resolution, 32 scans, KBr method) in the laboratory of Fibre and Biocomposite Development Centre (FIDEC) located in Banting, Selangor.

#### 2.3.2. Thickness Swelling (TS)

The particleboard samples were cut into sizes of 50 mm × 50 mm so that the thickness of the samples could be measured at the point where two diagonals intersects with 0.01 mm precision using vernier calipers. The samples were submerged in clean water. The thickness of the samples was then re-measured after soaking for 2 h and 24 h. The thickness swelling was then calculated by using the formula as shown below:

TS (%) = (T_2_ − T_1_)/T_1_ × 100

where,

T_1_: Initial thickness of sample before immersion (mm)

T_2_: Thickness of sample after immersion for 2 h/24 h (mm)

#### 2.3.3. Water Absorption (WA) 

Water absorption test was determined with the percentage of increasing in the weight of the samples after completely immersing in water after a specific period of time. The particleboard samples were cut into sizes of 50 mm × 50 mm and later the weight of the samples was calculated first before immersing them in water. The weight of the samples was re-weighed after 2 h and 24 h of immersion. The data of each samples was collected and calculated by using the formula as shown below:

WA (%) = (W_2_ − W_1_)/W_1_ × 100

where,

W_1_: Initial weight of sample before water soaking (g)

W_2_: Weight after water soaking for 2 h/24 h (g)

#### 2.3.4. Mechanical Properties

Mechanical properties including internal bonding (IB), modulus of rupture (MOR) and modulus of elasticity were determined according to the procedures stipulated in JIS A 5908:2003 [8]. 

#### 2.3.5. Fungal Decay Resistance 

The fungal decay resistance test of the particleboard samples was evaluated according to ASTM D1413-76: Standard test method for wood preservatives by laboratory soil-block cultures [9]. White rot fungus *Pycnoporus sanguineus* was used in this study. The fungus was obtained from Forest Research Institute Malaysia, Kepong, and were cultured in Wood Deterioration and Treatment Laboratory at Faculty of Forestry and Environment, Universiti Putra Malaysia. For each group, 5 replicates of test blocks of 25 mm long × 25 mm wide were prepared. Each culture bottles were filled with 150 g sieved soil and 70 mL distilled water. For fungus inoculation, feeder strip using rubberwood (35 mm long × 28 mm wide × 3 mm thick) was placed on top of the soil in each culture bottles. Then, it was steam-sterilized at 121 °C for 30 min and cooled at room temperature. After cooling, *P. sanguineus* was placed at the corner of feeder strip and the bottles were incubated at temperature between 25 to 27 °C for approximately 3 weeks. When the feeder strips were covered by mycelium, test blocks were placed on top of the mycelium-covered feeder strips. Then, a 16-week of incubation for the bottles that were containing test blocks were started. At the end of the incubation period, the test blocks were taken out from the bottles and all the mycelium on the test blocks surfaces were removed. The test blocks were oven-dried until constant weights were attained. Each block was weighed and the percentage of weight loss for the test block was calculated using the equation below:

WL (%) = 100 (W_i_ − W_f_)/W_i_

where,

W_i:_ The initial weight of test block before exposure to fungi (g) 

W_f_: The weight of test block after exposure to fungi (g)

#### 2.3.6. Termite Resistance 

Termite resistance test was carried out based on ASTM D3345-08: Laboratory evaluation of wood and other cellulosic materials for resistance to termite [10]. Firstly, test blocks of 25 mm length × 25 mm width were cut from the particleboard samples. Subterranean termite, *Coptotermus curvignathus* was used in this test. They were collected from an infested pine plantation located in Institute of Bioscience, Universiti Putra Malaysia. The subterranean termites were trapped using pine wood blocks as bait. Then, they were separated from debris using bridge method. The test blocks were placed in a culture bottle filled with approximately 200 g of sterilized sand mixed with 30 mL of distilled water. About 1 ± 0.05 g of *C. curvignathus* were weighed and introduced into each bottle filled with sand. The ratio of workers: soldiers is 9:1. The cultured bottles were then covered with black plastic and left at room temperature for 4 weeks. At the end of the test period, the test blocks were removed and oven-dried until they reached constant weights. Each test block was examined based on percent weight loss using the equation as shown below:

WL (%) = 100 (W_i_ − W_t_)/W_i_

where,

W_i:_ The initial weight of test block before exposure to termites (g) 

W_t_: The weight of test block after exposure to termites (g)

### 2.4. Data Analysis

The data of both citric acid- and UF-bonded particleboard were collected and analysed using Statistical Analysis System (SAS) procedure for the analysis of variance (ANOVA) at 95% confident level (*p* ≤ 0.05). Besides, Tukey’s Honest Significant Difference (HSD) test was used to further determine the significant level of average values for the treatment.

## 3. Results and Discussion

### 3.1. FTIR Analysis 

**Figure 1:**
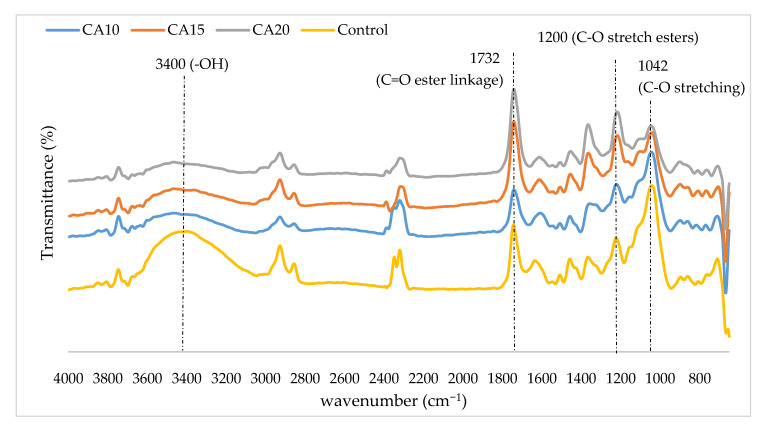
Fourier Transform Infrared (FTIR) spectra (4000 cm^−1^–600 cm^−1^) of control and rubberwood particles sprayed with different citric acid content. CA10 = 10 wt% citric acid, CA15 = 15 wt% citric acid, CA20 = 20 wt% citric acid.

The effects of citric acid content on the chemical structure of rubberwood particles are illustrated by the normalized spectra as shown in Figure 1. The functional groups for all of the samples are more or less the same, except for the intensity of some peaks. The peaks represent –OH groups at around 3400 cm⁻^1^ broadened for citric acid treated particles compared to that of the control rubberwood particles. The decrement in peak intensity implied reduction in free –OH in citric acid treated particles due to the formation of ester linkages between –OH on cellulose and carbonyl groups of citric acid [11]. Similar observation was also found at absorption peak near 1042 cm⁻^1^ which belongs to C–O stretching in cellulose I and cellulose II [12]. The peak intensity decreased along with increasing citric acid content. On the other hand, the peaks at around 1732 cm⁻^1^ correspond to the C=O stretching derived from carboxyl group and C=O ester linkage [13]. Notably, the peak intensity increased along with increasing citric acid content from 10 wt% to 20 wt%. In addition, the peak at around 1200 cm^−l^ also represent the C–O stretch esters [14]. The intensity of the peak also increased along with increasing citric acid content. These observations supported the fact that citric acid has reacted with the hydroxyl groups of rubberwood particles to form ester linkages and contribute adhesiveness to the particleboard. 

### 3.2. Thickness Swelling (TS) and Water Absorption (WA)

**Figure 2:**
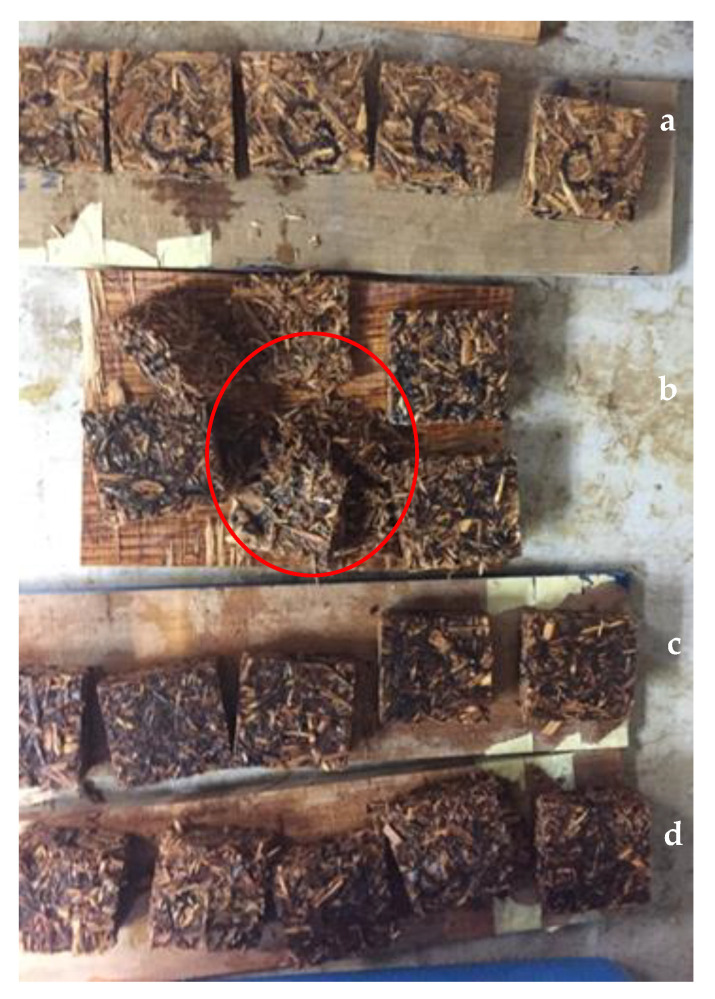
Visual appearance of particleboards after 24 h immersion in water; (**a**) control, (**b**) 10 wt% citric acid, (**c**) 15 wt% citric acid and (**d**) 20 wt% citric acid, where disintegration of particleboard (red circle) was noticed.

The density of the particleboards produced in this study ranged from 692.49 to 705.61 kg/m^3^. Table 1 listed the WA and TS of the particleboards after 2 h and 24 h water immersion. The WA_2h_ value of control UF-bonded particleboard was recorded as 90.57% while the WA_24h_ was 114.92%. However, citric acid-bonded particleboard exhibited significantly higher WA compared to that of control particleboard. As for citric acid-bonded particleboard, WA after 2 h was 145.3%, 148.03% and 120.89%, respectively for those bonded with 10, 15 and 20 wt%, respectively. Higher citric acid content (20 wt%) resulted in lower WA. After water immersion of 24 h, particleboard samples bonded with 10 wt% and 15 wt% experienced different extent of disintegration as some particles were felled off, as shown in Figure 2 during the immersion process. From Figure 2, it can be seen that the particleboards bonded with 10 wt% citric acid were disintegrated (indicated by red circle) after 24 h immersion in water. Particleboards bonded with 15 wt% citric acid experienced less severe disintegration compared to that of samples bonded with 10 wt%. This observation was not found in the control samples and samples bonded with 20 wt% citric acid, which indicated that they have good particle-particle adhesion. 

Thickness swelling of particleboard produced with UF and different citric acid content are shown in Table 1. After 2 h immersion, UF-bonded (control) particleboard recorded TS of 29.4% and further increased to 44.1% after 24 h immersion in water. In comparison, citric acid-bonded particleboard exhibited higher thickness swelling after 2 h and 24 h immersion. However, the thickness swelling decreased along with increasing citric acid content. Particleboard bonded with 20 wt% citric acid has significantly lower thickness swelling compared to that of the particleboard bonded with 15 wt% and 10 wt% citric acid. Particleboard bonded with 20 wt% citric acid reported 72.8% TS after 24 h immersion while those bonded with 15 wt% and 10 wt% recorded 92.2% and 154.4%, respectively. All the particleboard, even control samples, failed to meet the maximum allowable thickness swelling of 12% as stipulated in JIS A 5908. The findings were in agreement with Syamani et al. [15] who reported that *Imperata cylindrica* particleboards bonded with 20 wt% citric acid had the lowest TS compared to that of particleboard with 10 wt% and 15 wt%, respectively. As the ester linkages increased along with increasing citric acid content, the adhesiveness improved and subsequently led to improved TS [15]. This observation was also supported by the FTIR in Figure 1. Nevertheless, the findings suggested that citric acid-bonded particleboard exhibited significantly superior properties compared to that of the UF-bonded particleboard. One of the reasons for this observation might be excessive moisture after spraying with citric acid, especially in the case of 15 wt% and 20 wt% citric acid. Excessive moisture is reported to cause poor bonding as effective reaction between the adhesive and lignocellulosic materials was inhibited [16]. As for samples bonded with 10 wt% citric acid, the low content may not provide sufficient adhesiveness. 

### 3.3. Mechanical Properties

The effects of citric acid content on the mechanical properties of particleboard are shown in Table 2. Control particleboard bonded with UF resin gives IB value of 0.76 N/mm^2^. When citric acid was used as binding agent, inferior IB was observed as the IB of 0.24 N/mm^2^ was recorded in the particleboard bonded with 10 wt% citric acid. However, higher IB was recorded as the content of citric acid increased to 15 and 20 wt%. These IB values surpassed the minimum requirement for Type 8 particleboard as stipulated in JIS A 5908, which is 0.15 N/mm^2^. Similar trend was observed for MOR and MOE. Both MOR and MOE values of the particleboard bonded with UF resin was significantly higher than that of citric acid-bonded particleboard. The MOR and MOE values of the particleboard increased along with increasing citric acid content. Particleboard bonded with 20 wt% citric acid had significantly higher bending strength than that of the particleboard bonded with 10 wt% and 15 wt%. With the exception of control particleboard (9.27 N/mm^2^), particleboards bonded with citric acid did not met the minimum requirement for particleboard Type 8 as stipulated in JIS A 5908, where the bending strength shall be 8 N/mm^2^ or over. However, particleboard bonded with 20 wt% citric acid (7.66 N/mm^2^) are very close to the minimum requirement.

Kusumah et al. [14] reported the same findings in their study as the bending strength and IB of the particleboards increased along with increasing citric acid content. The authors also reported that the particleboard bonded with 20 wt% citric acid has lower MOR compared to that of PF- and pMDI-bonded particleboard. Nevertheless, higher IB values were recorded in citric acid-bonded particleboard. In addition, the values reported in their study are also higher than the present study. This phenomenon may be due to the particles used in Kusumah et al. [14] having undergone a pre-dried stage at 80 °C for 12 h after being blended with citric acid. Therefore, the particles had lower moisture than the current study, which might reduce the negative effect during hot pressing. Kelly [16] has pointed out that excessive moisture content bound to adversely affected the bondability of the particleboards by interfering the chemical reaction between adhesive and lignocellulose materials. Therefore, it is recommended that a pre-drying process should be carried out to improve the mechanical properties of the produced particleboard. Pre-drying is a process to reduce the moisture of the particles after sprayed with citric acid solution prior to hot pressing. As reported by Kusumah et al. [14] and Syamani et al. [15], sprayed particles dried at 80 °C for 12 h resulted in particleboard with better physical and mechanical properties. 

### 3.4. Fungal Resistance 

**Figure 3:**
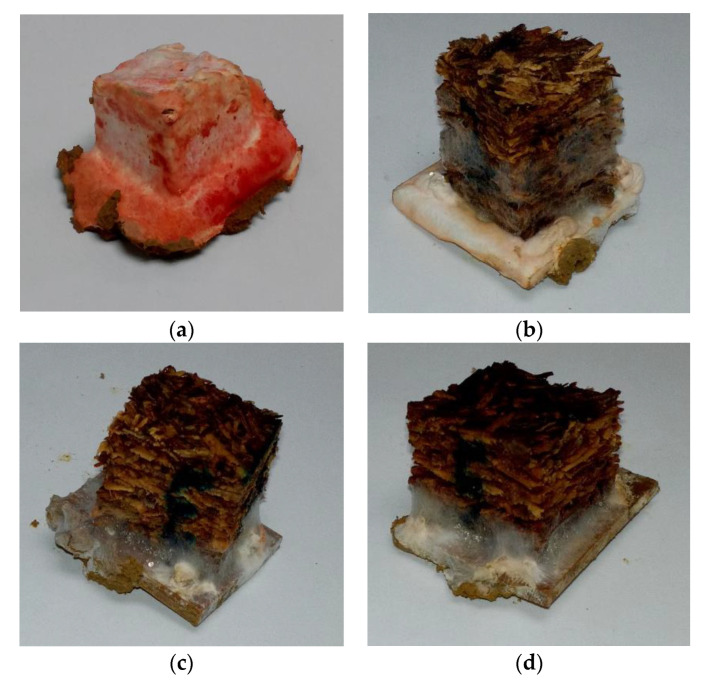
Visual appearance of particleboard (25 mm × 25 mm) after 16-week exposure to *Pycnoporus sanguineus*: (**a**) control *, (**b**) 10 wt% citric acid, (**c**) 15 wt% citric acid * and (**d**) 20 wt% citric acid. * Zhou et al. [17].

**Figure 4:**
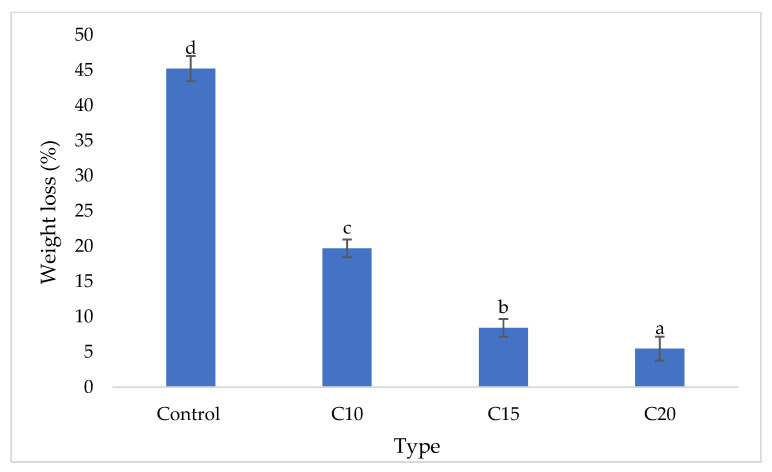
Weight loss of particleboard samples after 4-week exposure to *Pycnoporus sanguineus.* Note: Means followed by the different letters a, b, c and d are significantly different at *p* ≤ 0.05. CA10 = 10 wt% citric acid, CA15 = 15 wt% citric acid, CA20 = 20 wt% citric acid.

The visual appearance of control and particleboards bonded with different citric acid content after 16-week exposure to *Pycnoporus sanguineus* are exhibited in Figure 3. The control particleboards were fully covered by the mycelia of *Pycnoporus sanguineus*. In citric acid-bonded particleboard, the mycelia coverage is significantly lesser compared to that of control samples. The weight loss caused by white rot fungus are shown in Figure 4. After 16 weeks of exposure, control samples lost 45.22% of their original weight. On the other hand, particleboard bonded with 10, 15 and 20 wt% citric acid lost 19.71%, 8.40% and 5.45%, respectively. The weight loss trend suggesting that the resistance towards fungus increased as the citric acid content increased. 

Citric acid offers a good protection to wood towards fungal decay as a result of the cross-linking of citric acid and hydroxyl groups of the wood components [1]. Mubarok et al. [18] treated beech wood with 30% *w*/*w* sorbitol–citric acid (SorCA) aqueous solutions and reported that the treated wood is very durable against brown rot and white rot fungi. Essoua et al. [19] also reported the same observation where white pine and lodgepole pine treated with admixture of citric acid and glycerol. Larnoy et al. [20] impregnated pine sapwood with SorCA and found almost no decay evident by brown-rot fungi in the treated samples. The improvement in fungal resistance could be due to the acidic nature of citric acid itself. In addition, reduction of wood moisture content as a result of bulking effect in wood by citric acid could also contributed to the improvement in fungal resistance [18]. Compared to wood, investigation on the fungal resistance of citric acid-bonded wood composite is relatively scarce. Kusumah et al. [6] found that sweet sorghum bagasse particleboard bonded with 20 wt% citric acid had better resistance against white rot fungus, *Trametes versicolor*, compared to that of pMDI-bonded particleboard. 

### 3.5. Termite Resistance 

**Figure 5:**
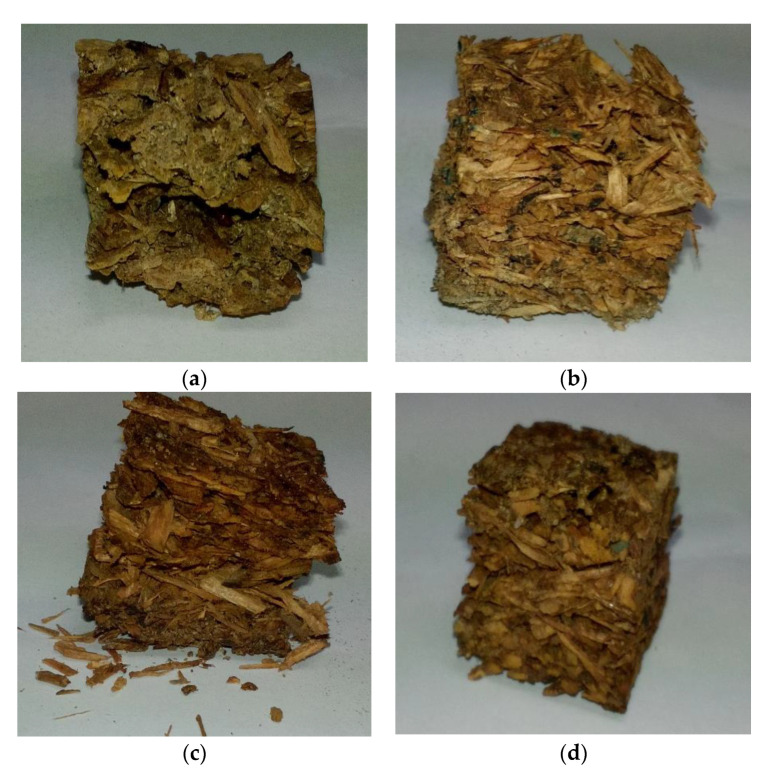
Visual appearance of particleboard (25 mm × 25 mm) after 4-week exposure to *Coptotermus curvignathus*: (**a**) control, (**b**) 10 wt% citric acid, (**c**) 15 wt% citric acid * and (**d**) 20 wt% citric acid. * Zhou et al. [17].

**Figure 6:**
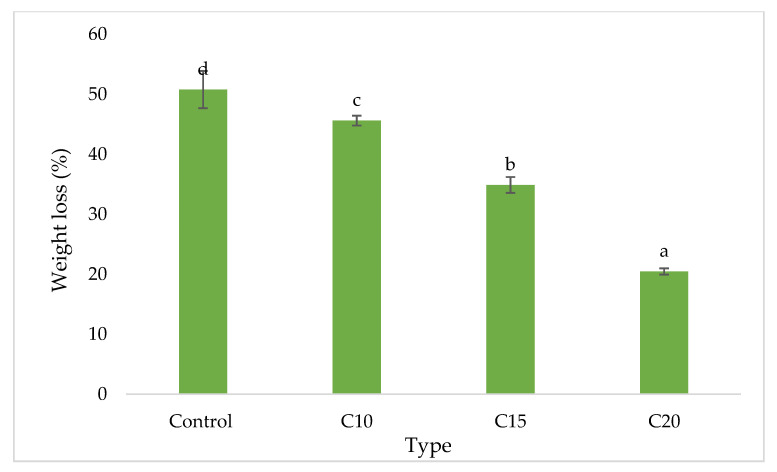
Weight loss of particleboard samples after 4-week exposure to *Coptotermus curvignathus.* Note: Means followed by the different letters a, b, c and d are significantly different at *p* ≤ 0.05. CA10 = 10 wt% citric acid, CA15 = 15 wt% citric acid, CA20 = 20 wt% citric acid.

Figure 5 illustrated the visual appearance of control rubberwood particleboard and particleboard bonded with different citric acid content after 4 weeks of exposure to *Coptotermus curvignathus*. It can be seen that the control particleboard samples experienced heavy attack. Similarly, although slightly better than that of control from visual inspection, particleboard bonded with 10 and 15 wt% citric acid also experienced heavy attack. However, particleboard bonded with 20 wt% citric acid has relatively intact appearance compared to the other samples as it is moderately attacked by the termites. The weight loss caused by termites after 4-week exposure are listed in Figure 6. The weight loss of the samples decreased significantly along with increasing citric acid content. Control samples recorded a weight loss value of 50.79% while samples bonded with 20 wt% citric acid recorded 20.46%. 

Citric acid has been reported as an effective natural wood preservative. Tarasin and Rattanapun [21] exposed *Melaleuca cajuputi* wood to Asian subterranean termite, *Coptotermes gestroi* Wasmann, and found that wood vacumm impregnated with 15% citric acid has the same effectiveness against termites compared to that of 1.5% boron. The authors concluded that the citric acid might affect the metabolism of *C. gestroi* in a similar way that boric acid did, as reported by Toyoshima et al. [22]. Other researchers attributed the effectiveness of citric acid to the natural adhesion of citric acid and its inhibitory properties against termites [6,7]. Kusumah et al. [6] reported that sweet sorghum bagasse particleboard bonded with 20 wt% citric acid has similar termite resistance to particleboard bonded with polymeric 4,4′-methylenediphenyl isocyanate (pMDI). However, higher termite mortality was observed in the citric acid-bonded samples. Due to its high acidity, medium density fibreboard (MDF) from pineapple leaves fibres bonded with citric acid experienced lower mass loss when exposed to subterranean termites, *Coptotermes formosanus* Shiraki [7]. 

## 4. Conclusions

The effects of different citric acid content on the physical, mechanical properties and biological durability of rubberwood particleboard were investigated. Generally, UF-bonded particleboard had superior properties compared to that of the citric acid-bonded particleboard. However, increasing citric acid content from 10 wt% to 20 wt% had significantly improved both the physical and mechanical properties of the particleboard. In term of fungal resistance, citric acid-bonded particleboard has significantly better resistance against white rot fungus. Citric acid-bonded particleboard also displayed significant superior termite resistance compared to that of UF-bonded particleboard. Although inferior to UF-bonded particleboard, potential has been shown by the citric acid-bonded particleboard, particularly of those bonded with 20 wt%, as it surpassed the minimum requirement for internal bonding strength and was very close to attaining the minimum bending strength requirement as stipulated in JIS A 5908. Further researches are needed to improve the performance of the citric acid-bonded particleboard. A pre-drying process on sprayed particles is worth investigating in future work. Apart from that, crosslinker such as sucrose could also be an option to enhance the properties of the particleboard.

## Figures and Tables

**Table 1:** Density, water absorption and thickness swelling of particleboard bonded with urea formaldehyde (UF) and different citric acid contents.

Type	Density	WA_2h_	WA_24h_	TS_2h_	TS_24h_
Control	692.49 ± 11.76a	90.57 ± 3.74a	114.92 ± 4.47a	29.43 ± 3.77a	44.08 ± 4.08a
CA10	699.99 ± 6.30a	145.3 ± 5.45c	disintegrate	136.18 ± 4.65d	154.42 ± 3.92d
CA15	700.83 ± 10.56a	148.03 ± 6.38c	disintegrate	91.07 ± 3.44c	92.2 ± 4.54c
CA20	705.61 ± 4.09a	120.89 ± 5.51b	137.93 ± 7.64b	65.97 ± 2.29b	72.75 ± 4.32b

Note: Values after ± are standard deviations. Within the same column, means followed by the different letters a, b, c and d are significantly different at *p* ≤ 0.05. CA10 = 10 wt% citric acid, CA15 = 15 wt% citric acid, CA20 = 20 wt% citric acid.

**Table 2:** Internal bonding (IB), modulus of rupture (MOR) and modulus of elasticity (MOE) of particleboard bonded with UF and different citric acid contents.

Type	IB	MOR	MOE
Control	0.76 ± 0.06a	9.27 ± 0.19a	1543 ± 68a
CA10	0.24 ± 0.05c	5.51 ± 0.22d	1026 ± 41d
CA15	0.32 ± 0.06c	6.44 ± 0.47b	1187 ± 66c
CA20	0.45 ± 0.07b	7.66 ± 0.30c	1355 ± 52b

Note: Values after ± are standard deviations. Within the same column, means followed by the different letters a, b, c and d are significantly different at *p* ≤ 0.05. CA10 = 10 wt% citric acid, CA15 = 15 wt% citric acid, CA20 = 20 wt% citric acid.
